# Bioaccumulation, sources and health risk assessment of polycyclic aromatic hydrocarbons in *Lilium davidii* var. *unicolor*

**DOI:** 10.1371/journal.pone.0301114

**Published:** 2025-02-06

**Authors:** Haixu Sun, Tianxiang Xia, Hongguang Cheng, Zhenzhen Wu, Qianding Cheng, Lu Lu, Chunbao Fu

**Affiliations:** 1 Beijing Key Laboratory for Risk Modeling and Remediation of Contaminated sites, Beijing Municipal Research Institute of Eco-Environmental Protection, Beijing, China; 2 College of Water Sciences, Beijing Normal University, Beijing, China; 3 School of Environment, Beijing Normal University, Beijing, China; 4 Beijing Orient Institute of Measurement & Test, Beijing, China; 5 Chinese Academy of Environmental Planning, Beijing, China; Kanazawa University, JAPAN

## Abstract

Dietary uptake is the main pathway of exposure to polycyclic aromatic hydrocarbons (PAHs). However, there is no data regarding the pollution and health risks posed by PAHs in *Lilium davidii* var. *unicolor*. We measured the concentrations of 16 PAHs in lily bulbs from Lanzhou; analyzed the bioaccumulation, sources, and pollution pathways of PAHs; assessed the influence of baking on PAH pollution in the bulb; and assessed the cancer risks associated with PAH exposure via lily consumption. The total PAH concentrations in raw bulbs were 30.39–206.55 μg kg^-1^. The bioconcentration factors of total PAHs ranged widely from 0.92 to 5.71, with a median value of 2.25. Pearson correlation analysis revealed that the octanol-water partition coefficients and water solubility values played important roles in the bioaccumulation of naphthalene, fluorene, phenanthrene, pyrene, and fluoranthene in the raw bulb by influencing PAH availability in soil. Correlation analysis and principal component analysis with multivariate linear regression indicated that biomass and wood burning, coal combustion, diesel combustion, and petroleum leakage were the major sources of PAHs in the raw bulbs. The paired t-test showed that the PAH concentrations in the baked bulbs were higher than those in the raw bulbs. PAH compositions in lily bulb changed during the baking process. Baked bulbs exhibited a higher cancer risk than raw bulbs. Local adults had low carcinogenic risks from consuming lily bulbs. This study fills the knowledge gap about PAH pollution and the related health risks of PAHs in the Lanzhou lily.

## Introduction

Polycyclic aromatic hydrocarbons (PAHs) are volatile hydrocarbons that consist of two or more fused aromatic rings [1]. Some PAHs are associated with mutagenic and/or carcinogenic effects [2, 3], such as lung cancer [4], reproductive problems [5], and cognitive development impairment [6]. Various PAHs are released into the environment from anthropogenic sources, such as petroleum combustion [7], coal combustion [8–10], biomass burning [11], and oil spills [12, 13]. In 2004, the total global emissions of 16 PAHs were 5.2×10^5^ t year^-1^, and China was the largest emitter (1.14×10^5^ t year^-1^) [14]. The total emissions of 16 PAHs were 1.0×10^5^ t in 2017 in mainland China [15]. Agricultural soils are the main sinks and sources of PAHs [16]. High levels of PAHs in agricultural soils have been reported worldwide [16, 17]. PAHs in agricultural soils can be taken up by crops through the soil-root and air-shoot routes [18, 19].

Food safety is a common concern in the aggregation of environmental pollutants. Dietary uptake is considered the principal mechanism of PAH exposure [4, 19]. Vegetables are one of the most consumed foods in daily diet, especially in Asia. The Fifth China Total Diet Study, conducted in 2009–2013, found that the consumption of vegetables by six different age groups accounted for 20.2–29.0% of the total food consumption in China [20]. Dietary exposure to PAHs through vegetable consumption has been highlighted because of the high consumption of vegetables and the serious health effects of PAHs [21]. During food processing, cooking food at high temperatures generates free radicals [22]. The free radicals are recombined into light PAHs, and then the heavy PAHs [22]. These PAHs can move to the hydrophobic food chain compartment and finally remain in the food that is rich in fat [22]. Additionally, the incomplete combustion of organic matter in fuels can produce PAHs, leading to the contamination of food products [23, 24]. These processes lead to PAH pollution of food products. Therefore, it is important to analyze the influence of food processing on PAH pollution in vegetables.

*Lilium davidii* var. *unicolor* is a specialty of Lanzhou, Gansu province, and “Lanzhou lily” was officially approved as a national geographical indication product of China for it by the General Administration of Quality Supervision, Inspection, and Quarantine in September 2004. Lanzhou lily has developed into one of the characteristic agricultural pillar industries in Gansu Province [25]. *Lanzhou lily* is a bulb vegetable that contains a variety of nutrients [26–28]. The average soluble sugar, crude ash, and crude protein contents in *Lanzhou lily* are 236.20%, 58.72%, and 35.80% higher than those of other edible lilies, respectively, whereas the crude fiber content is 34.9% lower than that of other edible lilies [29]. Lanzhou lily is the only variety of edible sweet lily in China, which can be used for making porridge, making soup, steaming, stiring fried meat, and has been sold all over the country and abroad [30]. Lanzhou lily is a perennial herb, with the average growth cycle of six years, and is therefore prone to bioaccumulation of PAHs [31]. Mature lilies are usually consumed after cooking, and baking in a coal-fired furnace is one of the main cooking methods of Lanzhou lily used by the residents. Previous studies focused on the concentrations and potential health risks of metal(loid)s in Lanzhou lily [32, 33]. However, no research has been conducted on PAH contamination in the Lanzhou lily and the related health risks.

This study aimed to: (1) investigate the occurrence and bioaccumulation of 16 priority PAHs in Lanzhou lily; (2) analyze the sources of PAHs in the edible part of the lily; (3) analyze the influence of baking on PAH pollution in lily bulbs; and (4) assess the probabilistic health risks associated with lily consumption.

## Materials and methods

### Study area

Lanzhou is located in the central area of Gansu Province, Northwest China, and is characterized by a temperate continental climate. Due to the favorable climate, the Lanzhou lily has become one of the most distinctive agricultural products in mountainous areas with formidable natural conditions in Lanzhou [31, 34–36]. The Qilihe and Xigu Districts, which are the main planting bases of the Lanzhou lily, were selected as the study areas. In this area, the elevation is approximately 1700–2700 m. The annual average temperature is 8.5–8.9°C. The annual average frost-free period exceeds 180 days. The annual average duration of sunshine is 2446 h. The annual average rainfall is 300–400 mm. Additionally, it was estimated that as of 2020, the lily growers amounted to 11 000 households, the lily planting area reached 3467 hm^2^, the lily production reached 28,000 tons, the lily sales output value reached 860 million yuan, and the per capita lily income reached 8,996 yuan, accounting for 41% of the region’s farmers’ per capita net income in Qilihe District [37].

### Sample collection and analysis

#### Sample collection and preparation

Sample collection was conducted from October 1 to 8, 2018. Nine sampling points were selected from Xigu and Qilihe districts. At each sampling point, at least five lily bulb subsamples were collected using a stainless steel shovel and then mixed as one lily bulb sample. At the same time, agricultural soil samples (0–20 cm) were collected at five of these sampling points using a stainless steel soil sampler, and one soil sample was composed of at least five soil subsamples.

The bulbs were isolated from lily plants and washed three times with tap water and ultrapure water in sequence [32]. Each washed bulb sample was divided into two equal parts using the method of quartering [32]. One sample was freeze-dried, ground, and then sieved with a 0.150 mm nylon sieve, and the other sample was used to analyze the influence of baking on PAH contamination in bulbs. Soil samples were also freeze-dried, ground, and sieved with a 0.150 mm nylon sieve for the measurement of PAH concentrations.

To analyze the influence of baking on PAH pollution in the bulb, a coal-fired furnace and chunk coal, which are commonly used in rural areas of Lanzhou for heating and cooking, were selected. The real-life baking process was simulated, and the washed bulb samples were baked for about 15 minutes in the oven of the furnace when the chunk coal burned in the furnace under flaming conditions. During the baking process, the gaseous and particulate phases emitted from the coal combustion were collected at the outlet of the chimney using an air sampler (BUCK LIBRA PLUS LP-5) [38–45]. The flow rate of the air sampler was set at 1.5 L min^-1^. Quartz fiber filters (QFFs) (Munktell MK360, Sweden, *φ* 47 mm, retention efficiency < 99.95% for particles smaller than 0.3 μm) and polyurethane foams (PUFs) were used for the collection of the particulate and gaseous phase PAH samples, respectively. Before sampling, the PUFs were pre-extracted sequentially using acetone, dichloromethane, and hexane. Each extraction step was conducted for 8 h. The cleaned PUFs were wrapped in clean aluminum foil, which was wiped with acetone before use. The QFFs were prebaked at 450°C for 6 h to avoid interference from organic impurities, wrapped in aluminum foil, balanced in a dryer for 24–48 h, and weighed for use. After sampling, the PUFs were packed in aluminum foil and stored at -20°C before analysis. The QFFs containing PMs were wrapped in clean aluminum foil, balanced in a dryer, weighed, and stored at -20°C for further analysis. The baked bulb samples were freeze-dried, ground, and sieved with a 0.150 mm nylon sieve for PAH analysis.

#### Sample extraction and cleanup

Freeze-dried lily bulb samples were subjected to accelerated solvent extraction (ASE300, Dionex, USA). Samples were placed in a 33 ml ASE extraction tank and extracted with an n-hexane/dichloromethane (1/1) solution. The temperature was ramped up to 100°C for 5 min and then held at 100°C for 5 min. Extraction was conducted twice, followed by purging for 60 s. After extraction, the solution was cooled to room temperature and concentrated to approximately 2 ml using a rotary evaporator (R210/v700/v850, Buchi, Switzerland) with the water bath temperature set at 45°C and the vacuum degree at 250 hPa. Freeze-dried soil samples (5 g), particulate-loaded QFFs, and PUFs were extracted using the same method as the bulb sample.

Before cleanup, the Florisil column (Supelco, USA) was washed with 10 mL of a 2% acetone/n-hexane solution and then activated with 10 mL of n-hexane. The sample solution to be purified was transferred to an activated Florisil column for clean-up. When the liquid level dropped to 1 mm from the solid surface, 12 mL of 2% acetone/n-hexane (v/v) solution was used as the elution solvent, and the flow rate was controlled at approximately one drop per second. The eluate was concentrated to 1 mL using a parallel evaporator (CVE3100, Eyala, Japan) at a temperature of 50°C, degree of vacuum of 190 hPa, and rotating speed of 300 rpm. The concentrated solution was spiked with the internal standards for PAH analysis.

#### PAH analysis

PAH concentrations were analyzed using a gas chromatograph coupled with a mass spectrometer (GC-MS, QP2010, single quadrupole detector, Shimadzu, Japan), equipped with a DB-5MS capillary column (30 m × 0.25 mm × 0.25 μm). The oven temperature was programmed to be 70°C for 1 min, increased to 130°C at a rate of 20°C min ^-1^, to 210°C at 5°C min ^-1^, and then to 300°C for 4 min. Helium was used as the carrier gas. The parameters of GC/MS is given in S1 Table in S1 File. Detailed information on the target PAHs is presented in S2 Table in S1 File.

### Quality assurance and quality control

Quality assurance and quality control were provided by duplicate samples, recovery of surrogate compounds, and analytical blanks. One duplicate sample was used for each batch of bulbs, soil, QFFs, and PUFs samples, and the relative deviations were ≤ 50%. When the target PAH content in the samples was less than three times the detection limit, the relative deviation was ≤ 60%. Fluoranthene-d10 was used as a surrogate. Recoveries were 85%–115% for the soil, QFFs, and PUFs samples, and 70%–130% for the lily bulb sample. Analytical blanks were used as controls at each step of the analysis, and the measured values of the blank samples were all below the detection limits. The detection limits of PAHs in the samples are shown in S3 Table in S1 File.

### Statistical analysis

Statistical analyses were performed using SPSS 22.0. The undetected values of PAH concentrations were replaced by half of the detection limits when the detection rates were higher than 50%, whereas the undetected values of PAH concentrations were assumed to be zero when the detection rates were less than 50%. The Shapiro-Wilk method was used to determine the data distribution type. PAH concentrations in bulb samples were described using the arithmetic mean, standard deviation, median, coefficient of variation, maximum, and minimum. The paired t-test was used to analyze the difference between the mean PAH concentrations in raw bulb samples and those in baked bulb samples. Pearson correlation analysis was used to determine the correlation between PAH concentrations. Principal component analysis with multivariate linear regression (PCA-MLR) was used to analyze the sources of PAHs in the raw lily bulbs. The statistical significance level of the two-tailed test was *P* < 0.05.

### Bioconcentration factor of PAHs

The bioconcentration factor (BCF) represents the efficiency at which plants accumulate PAHs from the soil into their tissues. The BCFs of PAHs for the lilies were calculated using Eq (1):

$$BCF=\frac{C_{bulb}}{C_{soil}}$$ (1)

where C_bulb_ is the PAH concentration in the raw lily bulb in μg kg^-1^, C_soil_ is the PAH concentration in soil in μg kg^-1^, and BCF is the bioconcentration factor of PAHs from the agricultural soil to the lily bulb [46].

### PCA-MLR

PCA uses the method of "dimensionality reduction" to transform multiple variables into a smaller number of new variables (principal components). Based on the load of the principal components on each PAH component, the PAH pollution sources reflected by the principal components are inferred.

The purpose of MLR is to further determine the contribution rate of different pollution sources through the least squares method on the basis of source identification. The basic equation is:

$$y=\sum_{1}^{p}m_{i}X_{i}+b$$ (2)

where y represents the total concentrations of 16 PAHs, μg kg^-1^, p represents the number of the extracted principal components, X_i_ represents the factor score variable obtained from PCA, and b represents the remaining variable that has not been explained by the principal components. The independent and dependent variables are standardized, and the regression analysis is performed using Eq (3):

$$Z=\sum_{1}^{p}B_{i}X_{i}$$ (3)

where B_i_ is the coefficients of MLR. The contribution rate of source i can be calculated by Eq (4):

$$Contribution\;Rate_{i}(\%)=(B_{i}/\sum B_{i})\times 100\%$$ (4)

### Dietary exposure and cancer risks

The carcinogenic risks of the 16 PAHs are usually assessed using the total BaP equivalent concentration, which can be calculated using Eq (5) [47]:

$$BEC=\sum C_{i}\times TEF_{i}$$ (5)

where BEC is the total BaP equivalent concentration of 16 priority PAHs in bulb samples (μg kg^-1^, fresh weight), C_i_ is the content of *i*th PAH in the lily bulb (μg kg^-1^, fresh weight), and TEF_i_ is the toxic equivalence factor of *i*th PAH. TEFs are provided in S2 Table in S1 File.

The daily dietary PAH exposure dose (E_D_) was calculated using Eq (6) [48]:

$$E_{D}=\frac{BEC\times IR}{BW}$$ (6)

where IR is the ingestion rate of the lily bulb per day (g day^-1^) and BW is the body weight (kg).

The incremental lifetime cancer risk (ILCR) related to dietary exposure to PAHs via lily bulb consumption can be calculated using Eq (7):

$$ILCR=E_{D}\times EF\times ED\times SF\times CF/AT$$ (7)

where EF is the exposure frequency (days year^-1^), ED is the exposure duration (years), SF is the oral cancer slope factor of BaP ((mg kg^-1^ day^-1^)^-1^), CF is the conversion factor (1 × 10^-6^), and AT is the average lifespan (days). Probabilistic health risk assessment was conducted with a Monte Carlo simulation using Crystal Ball 11.1.2.4. The parameters and their distribution types are listed in S4 Table in S1 File.

If the ILCR value is < 1.0 × 10^−6^, the carcinogenic risk is considered to be at a safe level; if the ILCR value is in the range of 1.0 × 10^−6^ to 1.0 × 10^−4^, the carcinogenic risk is considered to be at a low level; if the ILCR value is > 1.0 × 10^−4^, the carcinogenic risk is considered to be serious [49, 50].

## Results and discussion

### Concentrations and composition of PAHs in raw lily bulbs

Among the 16 PAHs, the detection rates of Naphthalene (Nap), Acenaphthylene (Acy), Acenaphthene (Ace), Fluorene (Flu), Phenanthrene (Phe), Anthracene (Ant), Fluoranthene (Flt), and Pyrene (Pyr) were 100%, while BaA and Chr were detected in only one sample, and no 5-ring and 6-ring PAHs were detected. This is because the availability and uptake rate of PAHs decreases with increasing the number of benzene rings [51]. The PAH concentrations in the raw lily bulbs are shown in Table 1 and S5 Table in S1 File. The concentrations of PAHs in bulbs varied greatly, with the coefficients of variance of 40.70–97.91%. The total concentrations of 16 priority PAHs in bulb ranged from 30.39 μg kg^-1^ to 206.55 μg kg^-1^, with a median value of 154.66 μg kg^-1^ (S1 Fig in S1 File). The bulb sample with the highest total concentration of PAHs was collected from the point which was in the downwind direction of the petrochemical industry area. This can be attributed to the fact that large amounts of PAHs are emitted from industrial production and traffic. Additionally, PAHs can spill out during the storage and transportation of petroleum and derivative products [52, 53]. The concentrations of L-PAH in bulb ranged from 20.66 μg kg^-1^ to 178.35 μg kg^-1^, with a median value of 132.71 μg kg^-1^, and those of L-PAH in bulb ranged from 5.58 μg kg^-1^ to 59.79 μg kg^-1^, with a median value of 16.53 μg kg^-1^. The average proportion of L-PAH, H-PAH in lily bulb were 83.54% and 16.46%, respectively, which is consistent with existing research results [17, 52, 54–58]. Compared with other bulb vegetables (S6 Table in S1 File), the total 16 PAHs concentrations in the lily bulb tested in this study (145.49 μg kg^-1^) were obviously higher than those in garlic bulbs and onion bulbs from rural and urban areas in Romania [56]; and obviously higher than those in garlic bulbs and onion bulbs from Spain [59]; however, they were lower than those in onion and garlic bulbs from Pakistan [17]. Compared with other types of vegetables, the total 16 PAHs concentrations in lily bulbs obtained in our study (145.49 μg kg^-1^) were substantially higher than those found in Beijing (10.6–18.0 μg kg^-1^ for root vegetables; 30.1–47.4 μg kg^-1^ for leaf vegetables, fresh weight) [21]; significantly higher than those found in Shenzhen (2.81–66.0 μg kg^-1^, fresh weight) [60]; and significantly higher than those found in Catalonia, Spain (0.73–4.08 μg kg^-1^, fresh weight) [59]. This might be because the Lanzhou Lily has a long growth cycle of approximately six years [61], causing more PAHs to be bioaccumulated in lilies. However, the concentrations of PAHs determined in the bulb were substantially lower than those reported in Tianjin (340–850 μg kg^-1^) [62], and slightly lower than those found near the industrial areas of Shanghai (65.7–458.0 μg kg^-1^) [47], which may be attributed to the fact that the industrialization and urbanization of these two cities cause more PAH pollution in vegetables.

**Table 1:** Concentrations of PAHs in raw bulbs (n = 9).

PAHs	Ring number	Molecular weight g mol^-1^	Arithmetic mean μg kg^-1^, fw	Standard deviation	Median μg kg^-1^, fw	Minimum μg kg^-1^, fw	Maximum μg kg^-1^, fw	Coefficient of variance %
**Nap**	2	128.2	64.45	26.23	79.03	13.45	93.25	40.70
**Acy**	3	152.2	1.09	0.71	1.03	0.38	2.77	64.49
**Ace**	3	154.2	1.40	0.81	1.15	0.50	2.94	58.02
**Flu**	3	166.2	12.26	5.61	12.94	1.10	18.31	45.78
**Phe**	3	178.2	42.03	20.32	42.08	4.76	67.11	48.35
**Ant**	3	178.2	0.87	0.49	0.76	0.28	1.59	57.07
**Flt**	4	202.3	9.74	6.36	6.95	2.73	21.21	65.28
**Pyr**	4	202.3	12.69	10.95	9.58	0.13	31.57	86.26
**BaA**	4	228.3	1.63	1.59	1.63	0.50	2.75	97.91
**Chr**	4	228.3	2.63	2.30	2.63	1.00	4.26	87.59
**BbF**	5	252.3	-	-	-	-	-	-
**BkF**	5	252.3	-	-	-	-	-	-
**BaP**	5	252.3	-	-	-	-	-	-
**DahA**	5	278.4	-	-	-	-	-	-
**IP**	6	276.3	-	-	-	-	-	-
**BghiP**	6	276.3	-	-	-	-	-	-
**ΣPAHs**			145.49	58.36	154.66	30.39	206.55	40.11
**L-PAH**			122.11	47.94	132.71	20.66	178.35	39.26
**H-PAH**			23.38	18.04	16.53	5.58	59.79	77.16

fw, fresh weight; -, not detected in raw bulb sample; L-PAH, 2- and 3-ring PAHs; H-PAH, 4-, 5-, and 6-ring PAHs; ΣPAHs, the sum of the concentrations of 16 PAHs.

For individual PAH, the concentration of Nap was the highest, with the median value of 79.0 μg kg^-1^, ranging from 13.5 μg kg^-1^ to 93.2 μg kg^-1^, followed by Phe, Flu, Pyr, Flt, successively, with the median values of 42.1, 12.9, 9.6, and 6.9 μg kg^-1^, respectively. The composition of PAHs in the raw lily bulb samples is shown in S2 Fig in S1 File. 2-ring and 3-ring PAHs were predominant in all samples, accounting for 20.1–58.1% and 23.7–47.9%, respectively, and 4-ring PAHs account for 4.6–32.0%. This is because low molecular weight PAHs have greater water solubility and volatility, and high molecular weight PAHs are easily adsorbed by the organic matter in soil, which results in lower bioavailability of high molecular weight PAHs and consequently prevents them from being absorbed by plants [63–65].

### Bioaccumulation of PAHs from soil to bulb

**Fig 1:**
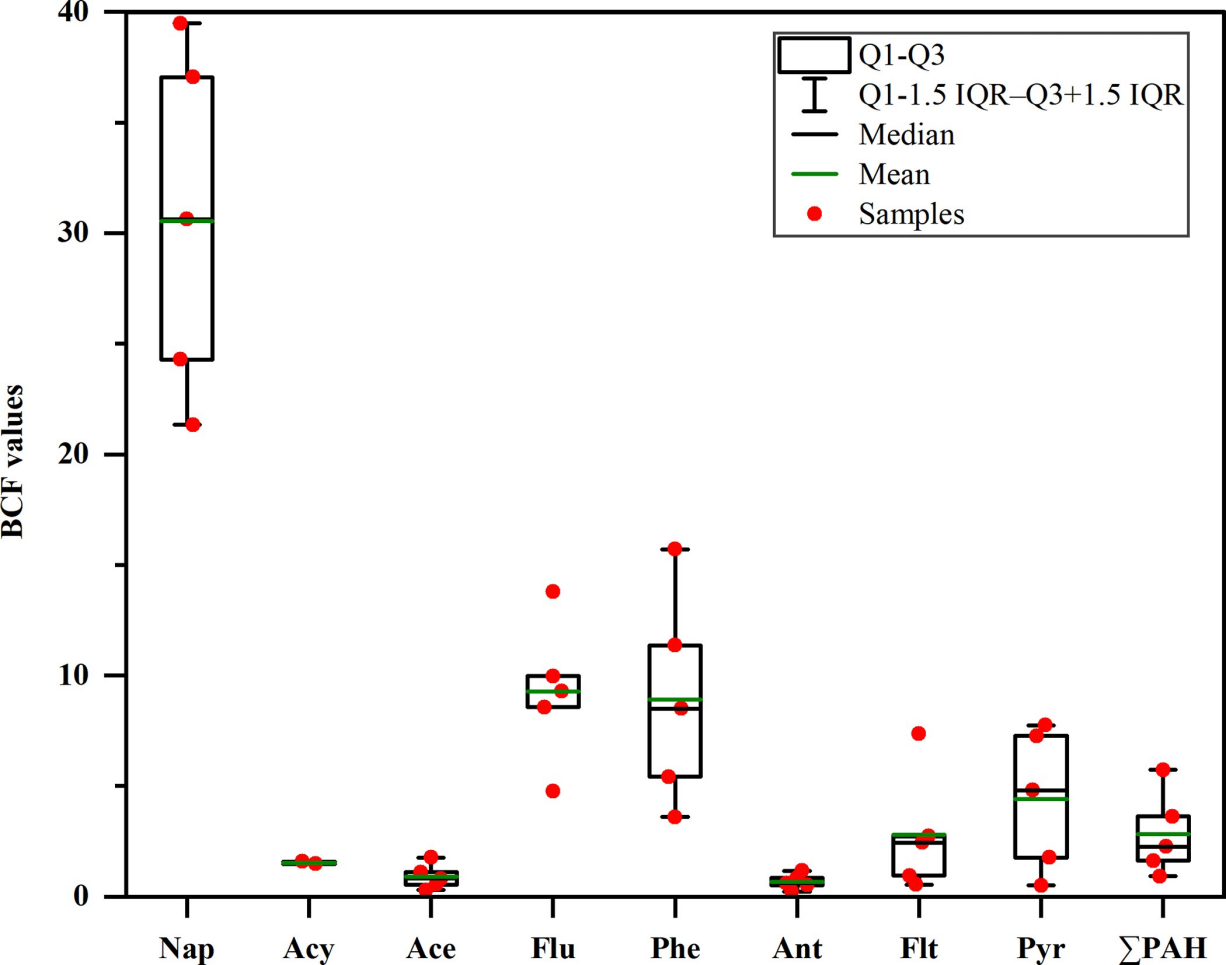
Soil-vegetable bioconcentration factors (BCFs) of PAHs for Lanzhou lily.

**Fig 2:**
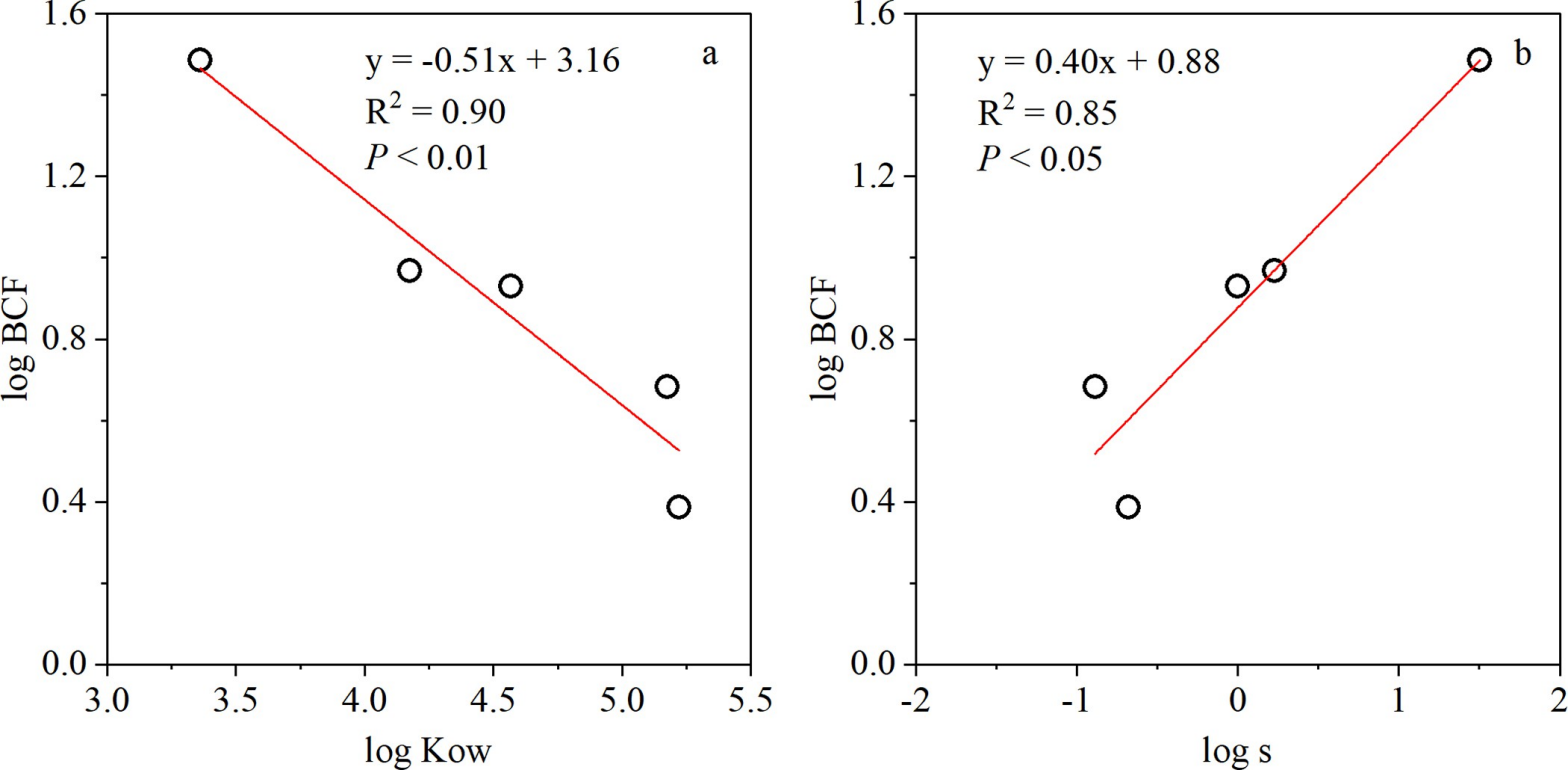
Correlation between the logarithm of the BCFs of Nap, Flu, Phe, Pyr, and Flt for lily and the logarithms of the octanol-water partition coefficient (Kow) (a) and the logarithms of solubility in water (S) (b) of Nap, Flu, Phe, Pyr, and Flt.

Based on the total concentrations of PAHs in the raw bulb (S5 Table in S1 File), the bulb samples numbered 1 to 5 were selected to analyze the bioaccumulation of PAHs from soil to bulb. The BCFs of the detected PAHs were calculated based on dry weight, and the results are shown in Fig 1 and S7 Table in S1 File. The BCFs of BaA and Chr are not shown in Fig 1, because they were detected in only one raw bulb sample. The soil-vegetable BCFs of PAHs in published studies are listed in S7 Table in S1 File. The BCFs of total PAHs in Lanzhou lily ranged widely from 0.93 to 5.73, with a median value of 2.26, which were higher than those for Chinese cabbage, lettuce, carrot, and cabbage, as reported by Chen et al. [66]. For individual PAH conger, the BCFs of Nap, Acy, Ace, Flu, Phe, Ant, Flt, and Pyr in Lanzhou lily were all higher than those in other bulb vegetables (onion and garlic) reported by Waqas et al. [17]. Moreover, the BCFs of Nap, Acy, Ace, Flu, Phe, Ant, Flt, and Pyr in Lanzhou lily were higher than those in other types of vegetables reported in previous studies (S7 Table in S1 File). Attention should be paid to PAH pollution in the Lanzhou lilies. This could be explained by the fact that vegetable physiology and soil characteristics were also determining factors for the differences in BCF values [17, 62, 66–67]. The mean values of the BCFs of individual PAHs decreased in the order of Nap > Flu > Phe > Pyr > Flt > Acy > Ace > Ant, with mean values of 30.55, 9.27, 8.92, 4.42, 2.81, 1.53, 0.91, and 0.68, respectively. Apart from the selective absorption by lily, the differences in BCFs might result from the different availabilities of PAHs in the soil. The octanol-water partition coefficient (Kow) and compound water solubility (S) are the two dominant factors affecting PAH availability in the soil. Kow indicates the adsorption tendency of the compound to the organic phase, whereas S indicates the compound’s mobility in the soil. In this study, the relationship between the BCF values of Nap, Flu, Phe, Pyr, and Flt in the lily bulb and the relevant Kow and S values were demonstrated by plotting logBCF against logKow and logS, respectively (Fig 2). Pearson correlation analysis showed that the logBCF values of Nap, Flu, Phe, Pyr, and Flt in the lily bulb were significantly negatively correlated with the corresponding logKow (*P* < 0.05) (Fig 2A) and logS values (*P* < 0.05) (Fig 2B). This demonstrates that PAHs with lower Kow values and higher S values (e.g., Nap, Flu, Phe, Pyr, Flt) are more easily bioaccumulated from the soil through the roots [68]. For example, Nap had the highest BCF value (30.62) because it had the lowest Kow (2.30 × 10^3^) and the highest S value (32.00 mg L^-1^) among the 16 priority PAHs.

### Sources and pollution pathways of PAHs in raw bulb

**Fig 3:**
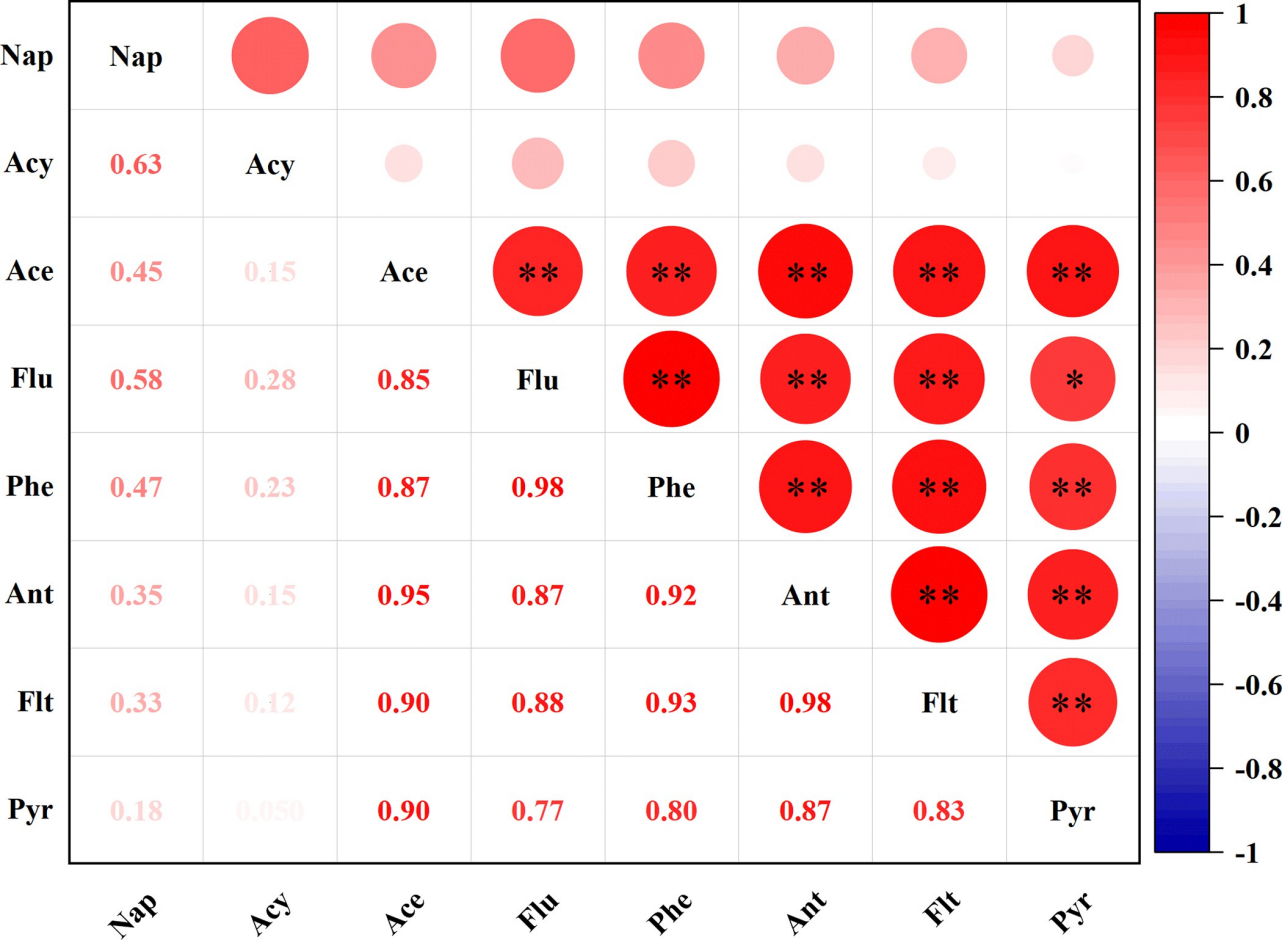
Pearson correlation analysis between the concentrations of PAHs in raw bulbs.

As shown in Fig 3, the Pearson correlation analysis showed that the concentrations of Ace, Flu, Phe, Ant, Flt, and Pyr in the raw bulb were generally significantly positively correlated with each other, indicating that these PAHs had similar pollution sources.

The PCA-MLR model was used to further investigate the possible sources of PAHs in the raw bulbs and the results are given in Table 2 and S8 Table in S1 File. Two factors (PC1 and PC2) were identified, accounting for 86.95% of the variance. PC1 was responsible for 62.70% of the variance and was dominated by Ace, Flu, Phe, Ant, Flt, and Pyr, which was consistent with the results of the correlation analysis. Ace, Flu, Phe, Ant, Flt, and Pyr are mainly emitted from biomass combustion [11, 69, 70], residential coal combustion [71–73], and industrial coal combustion [51, 74, 75]. In this study, biomass (wheat and corn stalks), wood, and coal were the main fuels and were widely used for heating and cooking in rural areas [76]. Lanzhou is an important industrial base in northwest China, and its industries include petrochemistry, energy and electric power, nonferrous metal smelting, and equipment manufacturing. Large quantities of PAHs are emitted from coal combustion in various types of industrial boilers and thermal power plants [77]. In this study, the sampling points were all close to the road, and Ace, Flu, Phe, and Ant have been reported to be predominant in diesel exhaust [78–81]. Thus, PC1 reflects the sources of biomass and wood burning, industrial and residential coal combustion, and diesel exhaust, which accounted for 57.97% according to the results of PCA-MLR. PC2 was responsible for 24.25% of the variance and was dominated by Nap and Acy. Nap and Acy originate from unburned petroleum [13, 80, 82, 83]. Lanzhou has the largest oil refining and chemical enterprises in western China. Therefore, the storage and transportation of petrochemical products and raw materials, and industrial production processes will lead to fugitive emissions of PAHs. For example, the highest concentration of Nap (93.25 μg kg^-1^) was determined in raw bulb sample collected from downwind of the petrochemical plant. Additionally, unburned petroleum can result from traffic emissions [80]. Therefore, PC2 indicated petroleum leakage, which accounted for 42.03%. Therefore, biomass and wood burning, coal combustion, diesel combustion, and petroleum leakage were the primary sources of PAHs in the raw lily bulbs. Based on the results of source analysis, it’s better to grow lilies in the areas away from the industrial areas, roads, and villages from the viewpoint of decreasing PAH health risk caused by lily digestion.

**Table 2:** Results of PCA-MLR.

Dependent variables	Independent variables	B ^a^	t ^b^	*P*-value ^b^	ΔR^2^ ^c^	B_i_/ΣB_i_ (%)
Standardization of the total concentrations of 8 PAHs	Standardization of the score variable of PC1	0.80**	13.57	0.00	0.64	57.97
	Standardization of the score variable of PC2	0.58**	9.88	0.00	0.34	42.03

^a^ The standardized estimates of regression coefficients.

^b^ t statistic and significance probability value of t test for independent variables.

^c^ Change of coefficients of determination.

* *P* value < 0.05

**P value < 0.01.

A correlation analysis was conducted to analyze the PAH contamination pathways in the raw bulb. Interestingly, the correlations between Flt concentration in edible parts and that in soil were negative for lily (S9 Table in S1 File), which was different from the findings for other types of vegetables by Jia et al. [47] and Tao et al. [62]. However, the mechanism underlying this phenomenon requires further investigation. The concentrations of Nap, Acy, Ace, Flu, Phe, Ant, and Pyr in the raw bulb were not significantly correlated with those in the soil, and the total PAH concentrations in the raw bulb were not significantly correlated with those in the soil (S9 Table in S1 File). There are two possible reasons for this observation. First, correlation analyses were conducted based on the total PAH concentrations in the soil, without considering the bioaccessibility of PAHs. Second, shoot uptake may also be an important pathway of Nap, Acy, Ace, Flu, Phe, Ant, and Pyr into the lily bulb, in addition to the soil-root route. PAHs with a Kaw > 10^−4^, including Nap, Acy, Ace, Flu, Phe, Ant, Flt, and Pyr, were reported to account for 98% of the gas absorption fluxes in shoots [84]. Additionally, PAHs with a Kaw > 10^−4^ were the major components in PAH fluxes from soil to air, and the soil-air-plant route plays an important role in the shoot uptake of PAHs with a Kaw > 10^−4^ [19, 84]. Therefore, shoot uptake may be a non-negligible pathway for PAH absorption by lily.

### Influence of baking on PAH contamination in bulbs

**Fig 4:**
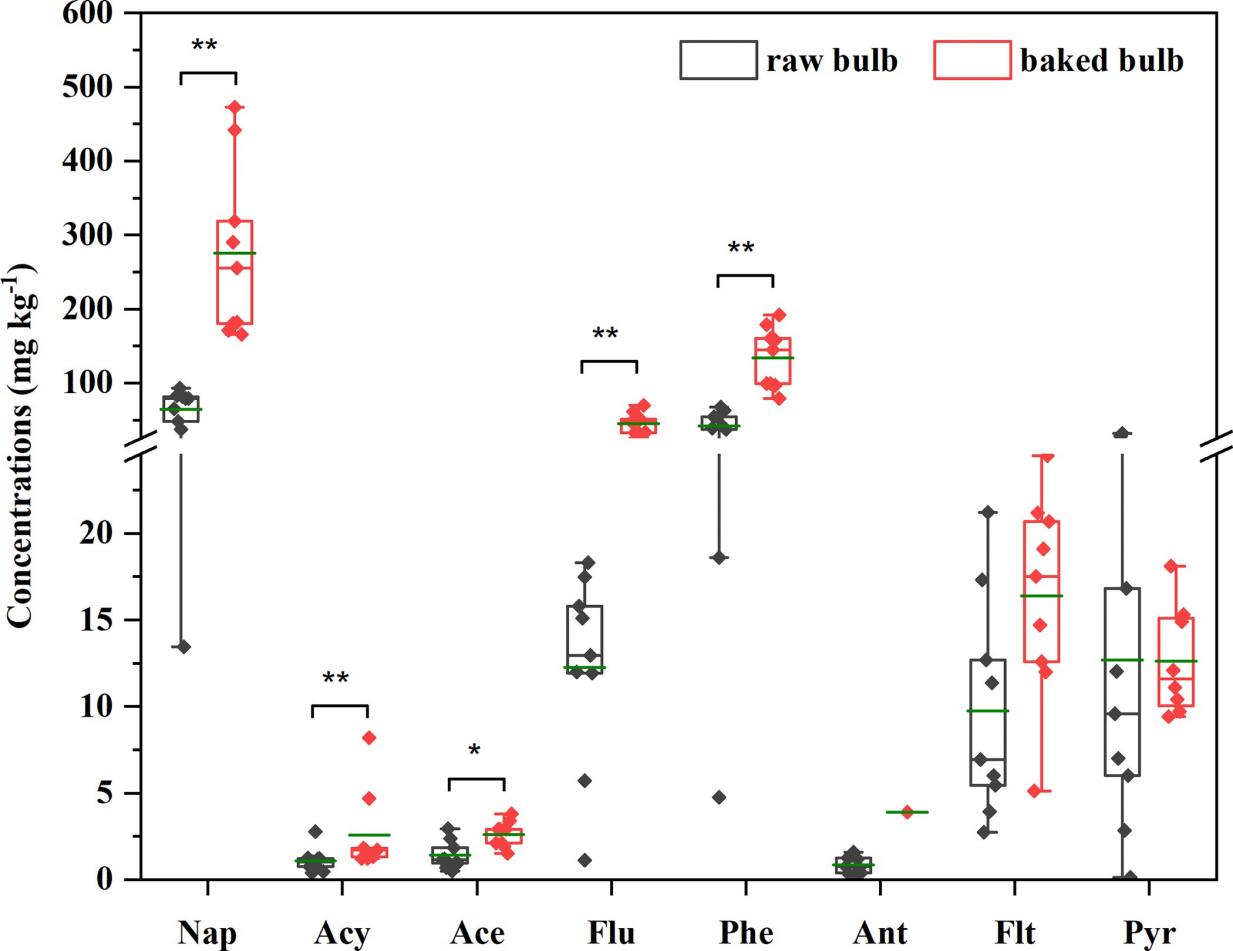
Concentrations of PAHs in raw bulbs and baked bulbs and the results of difference tests.

To analyze the influence of the combustion of the fuel on PAH contamination in bulbs during baking, the washed bulb samples were baked in the oven of the furnace when chunk coal burned in the furnace under flaming conditions. As shown in Fig 4 and S10 Table in S1 File, the total concentrations of 16 priority PAHs in baked bulbs ranged from 300.80 μg kg^-1^ to 743.00 μg kg^-1^, with the mean value of 488.51 μg kg^-1^, were higher than those in the raw bulbs. The paired t-test results showed that the concentrations of Nap (*P* < 0.01), Acy (*P* < 0.01), Ace (*P* < 0.05), Flu (*P* < 0.01), and Phe (*P* < 0.01) in the baked bulb were significantly higher than those in the raw bulb. Additionally, the concentrations of Flt and Pry in baked bulbs were higher than those in raw bulbs, although the difference was not statistically significant at the 0.05 significance level. Ant was detected in only one baked bulb sample; therefore, a difference test was not conducted for the Ant concentration. In general, as shown in S3 Fig in S1 File, Nap, Flu, Phe, Flt, and Pyr were predominant in the baked and raw bulbs. However, the contributions of Nap, Flu, Phe, and Ant to the baked bulbs were higher than those in the raw bulbs. The differences in PAH concentrations and compositions indicated that the bulbs were polluted during the baking process.

According to previous studies, there are two possible mechanisms for PAH pollution in baked foods. First, PAHs can be produced in plants by the pyrolysis of organic matter, mainly proteins, lipids, and steroids, during cooking [85]. Lanzhou lily is rich in protein, fat, soluble sugar, and starch [25, 37], which leads to the formation of PAHs during the baking process. Second, precursors are produced during the incomplete combustion of the fuel (e.g., coal), and these precursors can undergo a series of complex processes to form PAHs [23]. PAHs can be transported to the surface of the lily bulb together with smoke and then migrate inside [86].

In this study, the gaseous and particulate phases emitted from coal combustion were collected during the baking process, and the PAH profiles in the gaseous and particulate phases are shown in S4 and S5 Figs in S1 File. Considering the PAHs detected in bulbs, Phe, Ant, Flt, and Pyr were predominant in PM_2.5_ (S4a Fig in S1 File), and Phe, Flt, and Pyr in PM_10_ (S5A Fig in S1 File), which are emitted from coal combustion. In addition, it can be observed that Nap, Phe, and Flu were predominant in the gaseous phases (S4B and S5B Figs in S1 File). Therefore, coal combustion, especially the gaseous phases from chunk coal combustion, was the main source of PAH pollution in the bulb during baking. Additionally, the effect of the combustion of the fuel on PAH pollution in baked lily can also be demonstrated by the changes in the ratio of Flt/(Flt + Pyr) in the bulb. The ratios of Flt/(Flt + Pyr) in the baked and raw bulbs are shown in S6 Fig in S1 File. The mean value of the ratios of Flt/(Flt + Pyr) in baked bulbs was 0.54, which indicated that PAHs in baked bulbs were derived mainly from coal combustion [87]. Moreover, the mean value of the ratios of Flt/(Flt + Pyr) in baked bulbs was higher than that in raw bulbs (*P* > 0.05). This may be because the bulbs were polluted with PAHs emitted from coal combustion during the baking process. As shown in S6 Fig in S1 File, the mean values of the ratios of Flt/(Flt + Pyr) in the gaseous and particulate phases from chunk coal combustion were higher than those for the raw bulb. In addition, the mean value of the ratios of Flt/(Flt + Pyr) in the gaseous phases from the chunk coal combustion was higher than that for the baked bulb. Therefore, it can be concluded that emissions, especially the gaseous phases, from coal combustion contaminate the bulb during the baking process.

### Health risk assessment of PAHs via lily consumption

**Fig 5:**
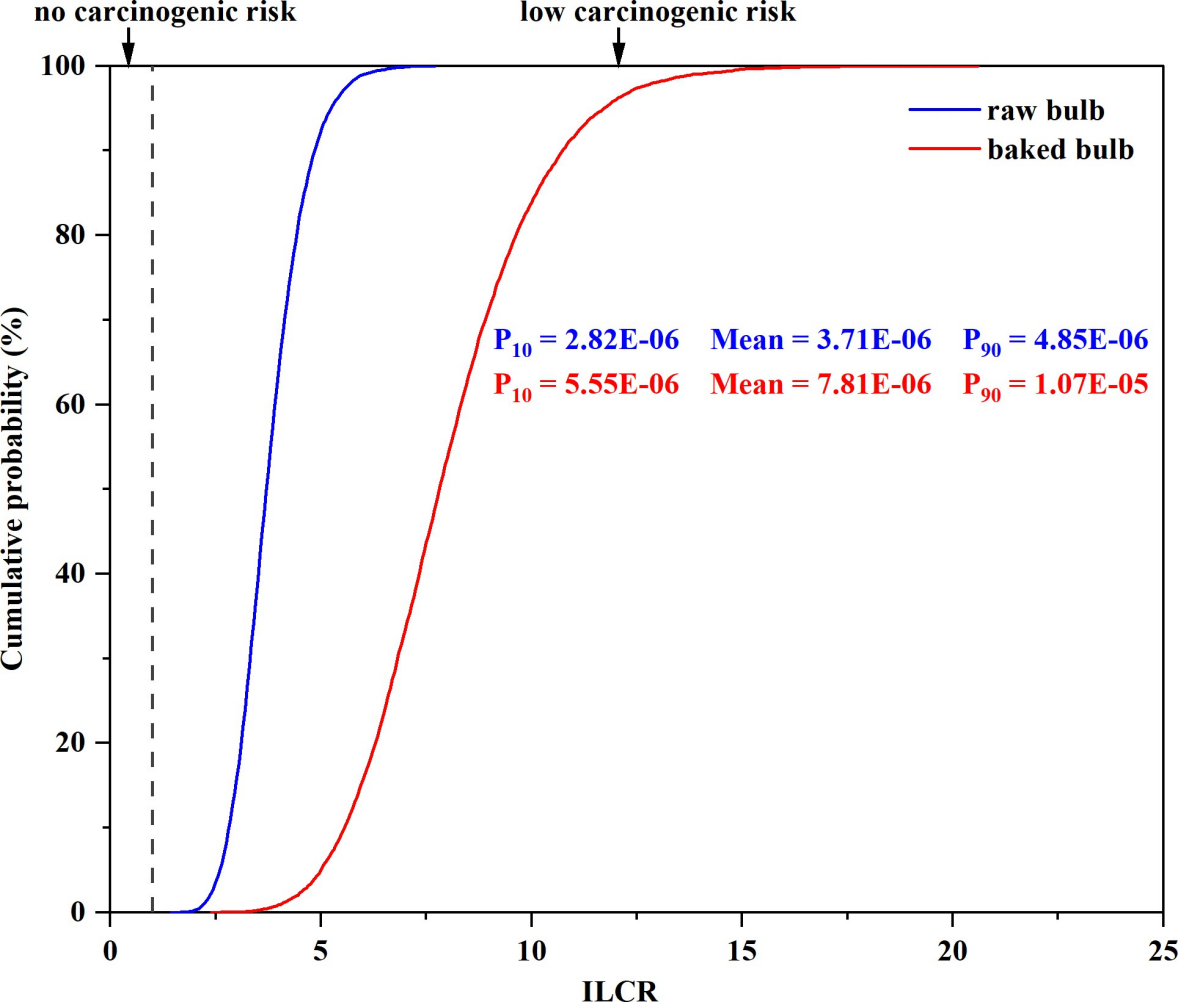
Incremental lifetime cancer risk (ILCR) values of PAHs in lily bulb before and after baking.

PAH cancer risks associated with ingesting raw bulbs and baked bulbs in local adults were assessed, and the results are shown in Fig 5. Baked bulbs exhibited higher cancer risks than raw bulbs, which might result from the higher concentrations of PAHs in baked bulbs than in raw bulbs. This indicates that PAH pollution resulting from baking caused higher cancer risks to the residents. The ILCR values for adults were between 10^-6^ and 10^-4^ via raw bulb and baked bulb consumption even at the 10th percentile, indicating that adults had low carcinogenic risks from consuming raw bulbs and baked bulbs. Baking in the oven of the furnace has been considered one of the main cooking methods of lily by residents, and cancer risks caused by exposure to PAHs via baked bulb consumption should be taken seriously.

The ILCR values for PAHs are given in S11 Table in S1 File. The carcinogenic risks decreased in the order of BaA > Nap> Phe > Chr > Pyr > Flu > Flt > Ant > Ace > Acy via raw bulb consumption, and in the order of Nap > BaA > Phe > Chr > Flu > Ant > Flt > Pyr > Acy > Ace via baked bulb consumption. Nap, BaA, Phe, and Chr were the major risk PAHs for lily, contributing more than 80% to the total carcinogenic risks. The ILCR values of BaA via raw bulb and baked bulb consumption and those of Nap and Phe via baked bulb consumption were all greater than 1.0 × 10^−6^ at the median level, indicating low carcinogenic risks.

## Conclusions

Two- and 3-ring PAHs were predominant in the raw bulbs. The BCFs of the total PAHs were higher than those of other bulb vegetables and other types of vegetables. Apart from the selective absorption by lily and characteristics of soil, the octanol-water partition coefficients and water solubility values played important roles in the bioaccumulation of Nap, Flu, Phe, Pyr, and Flt in the lily bulb by influencing PAH availability in soil. Biomass and wood burning, coal combustion, diesel combustion, and petroleum leakage were the major sources of PAHs in the raw bulbs. The concentrations of Nap, Acy, Ace, Flu, Phe, Flt, and Pyr were higher in the baked bulb than those in the raw bulb. Baked bulbs exhibited a higher cancer risk than raw bulbs did. Local adults had low carcinogenic risks from consuming baked bulbs and raw bulbs. PAH cancer risks associated with ingesting lily bulbs cooked in different ways in local adults could be assessed in future research. In this study, we analyzed the sources, pollution pathways, and health risks of PAHs in lily bulbs based on total PAH concentrations rather than bioaccessible PAH concentrations. Therefore, the bioaccessibility of PAHs must be considered in future studies. Because of the higher BCFs of PAHs for lily and PAH pollution introduced during the baking process, the cancer risks associated with PAH exposure via bulb consumption should be taken seriously.

## Supporting information

S1 File (ZIP)
