# Supplementary material for: Bioaccumulation, sources and health risk assessment of polycyclic aromatic hydrocarbons in Lilium davidii var. unicolor
Source: PLoS One. 2025 Feb 6;20(2):e0301114. doi: 10.1371/journal.pone.0301114 (PMC11801534; doi:10.1371/journal.pone.0301114)
Supplement: S1 File — (ZIP) [file pone.0301114.s001.zip › Supporting information.docx]

**Supplementary Materials**

***Bioaccumulation, sources and health risk assessment of polycyclic aromatic hydrocarbons in* *Lilium davidii* var. *unicolor***

Haixu Sun^1, 2^, Tianxiang Xia^1*^, Hongguang Cheng^2*^, Zhenzhen Wu^3^, Qianding Cheng^4^, Lu Lu^5^, Chunbao Fu^3^

^1^ Beijing Key Laboratory for risk modeling and remediation of contaminated sites, Beijing Municipal Research Institute of Eco-Environmental Protection, Beijing, 100037, China

^2^ College of Water Sciences, Beijing Normal University, Beijing, 100875, China

^3^ School of Environment, Beijing Normal University, Beijing, 100875, China

^4^ Beijing Orient Institute of Measurement & Test, Beijing 100094, China

^5^ Chinese Academy of Environmental Planning, Beijing, 100012, China

*Corresponding author:

E-mail address: [xiatianxiang@cee.cn](mailto:xiatianxiang@cee.cn) (TXX)

- **Table List:**

**S1 Table.** The parameters of GC/MS.

**S2 Table.** Individual target PAH information.

**S3 Table.** Detection limits of PAHs in samples.

**S4 Table.** Exposure parameters used for health risk assessment with the Monte Carlo simulator.

**S5 Table.** Concentrations of PAHs in each raw bulb sample.

**S6 Table.** Concentrations of PAHs in other bulb vegetables reported in published studies.

**S7 Table.** BCFs of PAHs for lily in this study and other types of vegetables in published studies.

**S8 Table.** Rotated factor pattern for PAHs in bulbs.

**S9 Table.** Pearson correlation coefficients between the concentrations of PAHs in bulbs and those in soil.

**S10 Table.** Concentrations of PAHs in baked bulbs.

**S11 Table.** Incremental lifetime cancer risk (ILCR) values of PAHs in lily bulb before and after baking.

- **Figure List:**

**S1 Fig.** The total PAH concentrations in each raw bulb sample.

**S2 Fig.** Composition of 16 PAHs in raw lily bulb samples.

**S3 Fig.** Comparison of detected PAHs in raw and baked bulbs.

**S4 Fig.** PAH composition profiles in PM_2.5_ and the corresponding gaseous phase from chunk coal combustion obtained by field investigation.

**S5 Fig.** PAH composition profiles in PM_10_ and the corresponding gaseous phase from chunk coal combustion obtained by field investigation.

**S6 Fig.** Diagnostic ratios of Flt/(Flt + Pyr) for raw bulbs, baked bulbs, and emissions from domestic coal combustion.

- **Reference**

**S1 Table.** **The parameters of GC/MS.**

| **GC/MS** | **Parameters** | **Analysis conditions** |
| --- | --- | --- |
| GC | chromatographic column | DB-5MS 30 m × 0.25 mm ID × 0.25 µm df |
|  | column temperature | 70 ℃ (1 min)–20 ℃ min^-1^–130 ℃–5 ℃ min^-1^–210 ℃–15 ℃ min^-1^–300 ℃ (4 min) |
|  | high purity helium | 1.0 mL min^-1^ (purity：99.999%) |
|  | pressure | 49.9 kPa |
|  | total flow | 44.9 mL min^-1^ |
|  | column flow | 2.32 mL min^-1^ |
|  | linear velocity | 55 cm s^-1^ |
|  | purging velocity | 10.0 mL min^-1^ |
|  | split ratio | 14.0 |
|  | injector temperature | 250 ℃ |
|  | injection method | Splitless (1 min) |
|  | injection volume | 2 µL |
| MS | ionization temperature | 230 ℃ |
|  | interface temperature | 270 ℃ |
|  | full scan range | m z^-1^ 35-500 |
|  | scan interval | 0.5 s |
|  | SIM sampling rate | 0.2 s |

**S2 Table.** **Individual target PAH information.**

| **English name** | **Abbreviation** | **Formula** | **Rings** | **MW**  **(g mol^-1^)** | **Pvap_25_**  **(Pa) ^a^** | **Kow ^b^** | **Sol_25_**  **(mg L^-1^) ^c^** | **logKaw ^d^** | **Carcinogenicity ^e^** | **TEF ^f^** |
| --- | --- | --- | --- | --- | --- | --- | --- | --- | --- | --- |
| Naphthalene | Nap | C_10_H_8_ | 2 | 128.2 | 10.4 | 2.3 × 10^3^ | 3.2 × 10^1^ | 0.56 | 2B | 0.001 |
| Acenaphthylene | Acy | C_12_H_8_ | 3 | 152.2 | 0.89 | 1.0 × 10^4^ | 3.9 | -0.75 | - | 0.001 |
| Acenaphthene | Ace | C_12_H_10_ | 3 | 154.2 | 0.29 | 8.3 × 10^3^ | 3.4 | -0.02 | 3 | 0.001 |
| Fluorene | Flu | C_13_H_10_ | 3 | 166.2 | 0.08 | 1.5 × 10^4^ | 1.7 | -1.06 | 3 | 0.001 |
| Phenanthrene | Phe | C_14_H_10_ | 3 | 178.2 | 0.025 | 3.7 × 10^4^ | 1.0 | -1.81 | 3 | 0.001 |
| Anthracene | Ant | C_14_H_10_ | 3 | 178.2 | 1.10 × 10^-3^ | 3.5 × 10^4^ | 4.5 × 10^-2^ | -1.40 | 3 | 0.01 |
| Fluoranthene | Flt | C_16_H_10_ | 4 | 202.3 | 1.10 × 10^-3^ | 1.66 × 10^4^ | 2.1 × 10^-1^ | -3.61 | 3 | 0.001 |
| Pyrene | Pyr | C_16_H_10_ | 4 | 202.3 | 5.50 × 10^-4^ | 1.5 × 10^5^ | 1.3 × 10^-1^ | -3.10 | 3 | 0.001 |
| Benzo[a]anthracene | BaA | C_18_H_12_ | 4 | 228.3 | 1.50 × 10^-5^ | 8.1 × 10^5^ | 5.7 × 10^-3^ | -5.48 | 2B | 0.1 |
| Chrysene | Chr | C_18_H_12_ | 4 | 228.3 | 6.10 × 10^-7^ | 7.2 × 10^5^ | 1.8 × 10^-3^ | -5.44 | 3 | 0.01 |
| Benzo[b]fluoranthene | BbF | C_20_H_12_ | 5 | 252.3 | 2.10 × 10^-5^ | 6.3 × 10^5^ | 1.4 × 10^-2^ | -6.12 | 2B | 0.1 |
| Benzo[k]fluoranthene | BkF | C_20_H_12_ | 5 | 252.3 | 1.30 × 10^-7^ | 1.0 × 10^6^ | 4.3 × 10^-3^ | -5.39 | 2B | 0.1 |
| Benzo[a]pyrene | BaP | C_20_H_12_ | 5 | 252.3 | 7.00 × 10^-7^ | 1.1 × 10^6^ | 3.8 × 10^-3^ | -7.91 | 1 | 1 |
| Dibenzo[a,h]anthracene | DahA | C_22_H_14_ | 5 | 278.4 | 4.30 × 10^-10^ | 5.6 × 10^6^ | 5.0 × 10^-4^ |  | 2A | 1 |
| Indeno[1,2,3-cd]pyrene | IP | C_22_H_12_ | 6 | 276.3 | 1.00 × 10^-10^ | 3.2 × 10^6^ | 5.3 × 10^-4^ |  | 3 | 0.1 |
| Benzo[ghi]perylene | BghiP | C_22_H_12_ | 6 | 276.3 | 1.40 × 10^-8^ | 3.2 × 10^6^ | 2.6 × 10^-4^ | -10.10 | 2A | 0.01 |

MW, molecular weight; Pvap_25_, the vapor pressure at 25 °C; Kow, octyl alcohol-water coefficient; Sol_25_, water solubility at 25 °C; Kaw, air-water partition coefficient.

^a^ [1]; ^b^ [1–2]; ^c^ [3]; ^d^ [4]; ^e^ The order of carcinogenicity of PAHs was 1 > 2A > 2 B > 3; ^f^ BaP equivalent toxicity coefficient [5].

**S3 Table.** **Detection limits of PAHs in samples.**

| **PAHs** | **QFFs sample**  **(ng)** | **PUFs sample**  **(ng)** | **Soil sample**  **(μg** **kg^-1^)** | **Bulb sample**  **(μg** **kg^-1^)** |
| --- | --- | --- | --- | --- |
| Nap | 25.0 | 25.0 | 2.8 | 1.3 |
| Acy | 4.0 | 4.0 | 0.4 | 1.0 |
| Ace | 3.7 | 3.7 | 0.4 | 0.9 |
| Flu | 3.9 | 3.9 | 0.4 | 1.0 |
| Phe | 5.6 | 5.6 | 0.6 | 0.4 |
| Ant | 1.4 | 1.4 | 0.2 | 0.4 |
| Flt | 3.7 | 3.7 | 0.4 | 0.9 |
| Pyr | 3.0 | 3.0 | 0.3 | 0.8 |
| BaA | 3.2 | 3.2 | 0.3 | 0.8 |
| Chr | 2.7 | 2.7 | 0.3 | 0.7 |
| BbF | 2.6 | 2.6 | 0.3 | 0.7 |
| BkF | 1.9 | 1.9 | 0.2 | 0.5 |
| BaP | 1.7 | 1.7 | 0.2 | 0.4 |
| DahA | 1.4 | 1.4 | 0.2 | 0.4 |
| IP | 1.4 | 1.4 | 0.2 | 0.4 |
| BghiP | 1.2 | 1.2 | 0.2 | 0.3 |

**S4 Table.** **Exposure parameters used for health risk assessment with the Monte Carlo simulator.**

| **Parameter** | **Description** | **Unit** | **Distribution type** | **Adults** | **References** |
| --- | --- | --- | --- | --- | --- |
| *IR* | ingestion rate of fruit vegetable | g day^-1^ | Point | 250 | [6] |
| *BW* | body weight | kg | Lognormal | LN (58.4, 10.8) | [7] |
| *EF* | Exposure frequency | day year^-1^ | Point | 365 | [8] |
| *ED* | exposure duration | years | Point | 24 | [9] |
| *SF* | oral cancer slope factor of BaP | (mg kg^-1^ day^-1^)^-1^ | Point | 7.3 | [8] |
| *AT* | average time of exposure to soil and vegetables | day | Point | 365*70 | [8] |

**S5 Table.** **Concentrations of PAHs in each raw bulb sample.**

| **PAHs** | **The number of the bulb sample** | | | | | | | | |
| --- | --- | --- | --- | --- | --- | --- | --- | --- | --- |
|  | **1** | **2** | **3** | **4** | **5** | **6** | **7** | **8** | **9** |
| Nap | 81.46 | 47.91 | 79.44 | 93.25 | 79.03 | 13.45 | 37.55 | 83.21 | 64.77 |
| Acy | 1.03 | 1.18 | 0.76 | 1.21 | 1.25 | 0.38 | 0.82 | 2.77 | 0.45 |
| Ace | 2.94 | 0.50 | 1.18 | 1.86 | 1.15 | 0.69 | 2.37 | 0.99 | 0.96 |
| Flu | 15.09 | 5.72 | 15.81 | 18.31 | 11.99 | 1.10 | 17.46 | 12.94 | 11.93 |
| Phe | 53.39 | 18.59 | 54.23 | 62.55 | 38.53 | 4.76 | 67.11 | 42.08 | 37.01 |
| Ant | 1.37 | 0.32 | 1.26 | 1.18 | 0.76 | 0.28 | 1.59 | 0.67 | 0.38 |
| Flt | 12.70 | 3.93 | 17.30 | 11.37 | 6.95 | 2.73 | 21.21 | 5.99 | 5.46 |
| Pyr | 28.27 | 2.82 | 12.03 | 16.83 | 9.58 | 7.00 | 31.57 | 5.99 | 0.13 |
| BaA | - | 0.50 | - | - | - | - | 2.75 | - | - |
| Chr | - | 1.00 | - | - | - | - | 4.26 | - | - |
| BbF | - | - | - | - | - | - | - | - | - |
| BkF | - | - | - | - | - | - | - | - | - |
| BaP | - | - | - | - | - | - | - | - | - |
| DahA | - | - | - | - | - | - | - | - | - |
| IP | - | - | - | - | - | - | - | - | - |
| BghiP | - | - | - | - | - | - | - | - | - |
| ΣPAHs | 196.25 | 82.48 | 182.02 | 206.55 | 149.24 | 30.39 | 186.69 | 154.66 | 121.09 |

-, not detected.

**S6 Table.** **Concentrations of PAHs in other bulb vegetables reported in published studies.**

| **Vegetables** | **Sampling areas** | **N** | **Statistical indexes** | **Nap** | **Acy** | **Ace** | **Flu** | **Phe** | **Ant** | **Flt** | **Pyr** | **BaA** | **Chr** | **BbF** | **BkF** | **BaP** | **DahA** | **IP** | **BghiP** | **ΣPAHs** | **Reference** |
| --- | --- | --- | --- | --- | --- | --- | --- | --- | --- | --- | --- | --- | --- | --- | --- | --- | --- | --- | --- | --- | --- |
| Garlic bulb | Urban area | 3 | AM ^a^ (μg kg^-1^) | 0.32 | 0.92 | 0.94 | 0.19 | 0.23 | ND | 0.92 | 0.92 | 0.92 | ND | - | 0.39 | 0.34 | 0.45 | 0.15 | 0.35 | 7.05 | [10] |
|  | Rural area | 3 | AM ^a^ (μg kg^-1^) | ND | 0.11 | 0.92 | 0.61 | 0.28 | 0.60 | 0.57 | 0.84 | 0.60 | ND | - | 0.54 | 0.57 | 0.11 | 0.24 | 0.60 | 6.60 |  |
| Onion bulb | Urban area | 3 | AM ^a^ (μg kg^-1^) | ND | 0.36 | 0.11 | 0.56 | 0.30 | 0.32 | ND | ND | ND | 0.56 | - | 0.18 | 0.22 | 0.24 | 0.36 | 0.36 | 3.57 |  |
|  | Rural area | 3 | AM ^a^ (μg kg^-1^) | 0.82 | 0.79 | 0.79 | 0.11 | ND | 0.45 | ND | ND | ND | ND | - | ND | ND | ND | ND | ND | 2.96 |  |
| Onion |  |  | AM ^a^ (μg kg^-1^) | <0.47 | <0.09 | <0.09 | <0.09 | <0.09 | <0.09 | <0.09 | <0.09 | <0.04 | <0.04 | <0.04 | <0.04 | <0.04 | <0.04 | <0.04 | <0.04 | 0.71 | [11] |
| Onion | high urbanized areas | 12 | AM ^a^ (μg kg^-1^) | 39.77 | 2.13 | 10.10 | 13.17 | 13.73 | 11.47 | 30.76 | 29.77 | 6.77 | 6.80 | 14.31 | 4.74 | 12.27 | 6.20 | 1.80 | 4.40 |  | [12] |
| Garlic |  | 12 | AM ^a^ (μg kg^-1^) | 26.80 | 1.07 | 7.13 | 9.43 | 33.33 | 8.90 | 35.21 | 28.50 | 7.35 | 7.37 | 9.27 | 0.57 | 11.30 | 1.10 | 2.30 | 1.55 |  |  |

^a^ arithmetic mean.

**S7 Table. BCFs of PAHs for lily in this study and other types of vegetables in published studies.**

| **Vegetables** | **Latin names** | **n** |  | **Nap** | **Acy** | **Ace** | **Flu** | **Phe** | **Ant** | **Flt** | **Pyr** | **BaA** | **Chr** | **BbF** | **BkF** | **BaP** | **DahA** | **IP** | **BghiP** | **∑PAHs** | **Reference** |
| --- | --- | --- | --- | --- | --- | --- | --- | --- | --- | --- | --- | --- | --- | --- | --- | --- | --- | --- | --- | --- | --- |
| Lily ^a^ | *Lilium davidii* var. *unicolor* |  | 1 | 37.05 |  | 1.76 | 9.96 | 8.51 | 0.85 | 2.44 | 7.75 |  |  |  |  |  |  |  |  | 2.26 | This study |
|  |  |  | 2 | 21.33 | 1.48 | 0.29 | 4.76 | 3.60 | 0.24 | 0.54 | 0.51 | 0.09 | 0.19 |  |  |  |  |  |  | 0.93 |  |
|  |  |  | 3 | 24.28 |  | 0.55 | 13.79 | 15.70 | 1.17 | 7.37 | 7.27 |  |  |  |  |  |  |  |  | 5.73 |  |
|  |  |  | 4 | 30.62 | 1.58 | 1.11 | 8.57 | 11.37 | 0.61 | 2.73 | 4.82 |  |  |  |  |  |  |  |  | 3.63 |  |
|  |  |  | 5 | 39.48 |  | 0.82 | 9.28 | 5.43 | 0.52 | 0.94 | 1.76 |  |  |  |  |  |  |  |  | 1.62 |  |
|  |  | 5 | AM ^g^ | 30.55 | 1.53 | 0.91 | 9.27 | 8.92 | 0.68 | 2.81 | 4.42 | 0.09 | 0.19 |  |  |  |  |  |  | 2.83 |  |
|  |  |  | SD ^h^ | 7.85 | 0.07 | 0.57 | 3.23 | 4.81 | 0.35 | 2.72 | 3.23 |  |  |  |  |  |  |  |  | 1.90 |  |
|  |  |  | median | 30.62 | 1.53 | 0.82 | 9.28 | 8.51 | 0.61 | 2.44 | 4.82 |  |  |  |  |  |  |  |  | 2.26 |  |
|  |  |  | CV ^i^ | 25.69 | 4.60 | 62.64 | 34.86 | 53.96 | 52.02 | 96.88 | 73.03 |  |  |  |  |  |  |  |  | 67.11 |  |
|  |  |  | min | 21.33 | 1.48 | 0.29 | 4.76 | 3.60 | 0.24 | 0.54 | 0.51 |  |  |  |  |  |  |  |  | 0.93 |  |
|  |  |  | max | 39.48 | 1.58 | 1.76 | 13.79 | 15.70 | 1.17 | 7.37 | 7.75 |  |  |  |  |  |  |  |  | 5.73 |  |
| lettuce ^b^ |  | 6 |  | 0.60 | 0.76 | 0.96 | 1.61 | 0.42 | 1.32 | 0.69 | 0.69 | - | - | - | - | - | - | - | - | 0.40 | [13] |
| cabbage ^b^ |  | 7 |  | 0.72 | 0.67 | 0.85 | 0.48 | 0.45 | 0.66 | 0.43 | 0.14 | - | - | - | - | - | - | - | - | 0.24 |  |
| chinese cabbage ^b^ |  | 10 |  | 3.01 | 0.23 | 0.28 | 0.54 | 0.56 | 1.39 | 0.61 | 0.23 | - | - | - | - | - | - | - | - | 0.42 |  |
| carrot ^c^ |  | 5 |  | 0.56 | 0.96 | 0.44 | 0.34 | 0.50 | 0.48 | 0.22 | 0.35 | - | - | - | - | - | - | - | - | 0.27 |  |
| cabbage ^b^ |  | 5 |  | 0.38 | 1.33 | 0.49 | 0.38 | 0.60 | 0.73 | 0.48 | 0.16 | - | - | - | - | - | - | - | - | 0.26 |  |
| chinese cabbage ^b^ |  | 9 |  | 1.99 | 0.32 | 0.39 | 0.48 | 0.67 | 1.64 | 0.71 | 0.26 | - | - | - | - | - | - | - | - | 0.46 |  |
| carrot ^c^ |  | 9 |  | 0.53 | 0.77 | 0.69 | 0.43 | 0.53 | 0.71 | 0.23 | 0.53 | - | - | - | - | - | - | - | - | 0.30 |  |
| onion ^a^ | Allium cepa | 12 | AM ^g^ | 0.55 | 0.07 | 0.53 | 0.60 | 0.19 | 0.56 | 0.64 | 0.13 | 0.30 | 0.30 | 0.60 | 0.27 | 0.56 | 0.17 | 0.64 | 0.35 |  | [12] |
|  |  |  | SD ^h^ | 0.15 | 0.05 | 0.39 | 0.20 | 0.03 | 0.04 | 0.21 | 0.04 | 0.21 | 0.21 | 0.28 | 0.32 | 0.08 |  |  |  |  |  |
| garlic ^a^ | Allium sativum | 12 | AM ^g^ | 0.34 | 0.04 | 0.38 | 0.73 | 0.50 | 0.60 | 0.46 | 0.12 | 0.22 | 0.28 | 0.54 | 0.08 | 0.68 | 0.07 | 0.15 | 0.17 |  |  |
|  |  |  | SD ^h^ | 0.07 | 0.02 | 0.10 | 0.15 | 0.36 | 0.21 | 0.15 | 0.06 | 0.16 | 0.06 | 0.20 | 0.05 | 0.06 |  |  | 0.02 |  |  |
| turnip ^c^ | Brassica rapa | 28 | AM ^g^ | 0.62 | 0.04 | 0.55 | 0.59 | 0.18 | 0.71 | 0.66 | 0.19 | 0.33 | 0.12 | 0.44 | 0.59 | 0.68 | 0.39 | 0.62 | 0.52 |  |  |
|  |  |  | SD ^h^ | 0.18 | 0.01 | 0.17 | 0.13 | 0.15 | 0.20 | 0.18 | 0.05 | 0.12 | 0.03 | 0.19 | 0.27 | 0.22 | 0.16 | 0.24 | 0.14 |  |  |
| carrot ^c^ | Daucus carota | 32 | AM ^g^ | 0.71 | 0.04 | 0.19 | 0.56 | 0.25 | 0.45 | 0.73 | 0.49 | 0.48 | 0.04 | 0.02 | 0.03 | 0.52 | 0.01 | 0.73 | 0.42 |  |  |
|  |  |  | SD ^h^ | 0.16 | 0.01 | 0.05 | 0.25 | 0.15 | 0.21 | 0.21 | 0.18 | 0.22 | 0.01 | 0.01 | 0.01 | 0.28 |  | 0.12 |  |  |  |
| radish ^c^ | Raphanus sativus | 30 | AM ^g^ | 0.66 | 0.03 | 0.17 | 0.65 | 0.44 | 0.61 | 0.66 | 0.37 | 0.45 | 0.63 | 0.58 | 0.15 | 0.65 | 0.42 | 0.85 | 0.68 |  |  |
|  |  |  | SD ^h^ | 0.17 | 0.01 | 0.06 | 0.15 | 0.23 | 0.18 | 0.15 | 0.24 | 0.19 | 0.25 | 0.29 | 0.04 | 0.25 | 0.31 | 0.94 | 0.14 |  |  |
| cabbage ^b^ | Brassica oleracea C. | 30 | AM ^g^ | 0.60 | 0.04 | 0.47 | 0.53 | 0.19 | 0.46 | 0.63 | 0.25 | 0.60 | 0.57 | 0.49 | 0.64 | 0.64 | 0.60 | 0.28 | 0.60 |  |  |
|  |  |  | SD ^h^ | 0.18 | 0.02 | 0.12 | 0.20 | 0.26 | 0.14 | 0.24 | 0.11 | 0.26 | 0.25 | 0.24 | 0.25 | 0.27 | 0.20 | 0.13 | 0.14 |  |  |
| lettuce ^b^ | Lactuca sativa L. | 15 | AM ^g^ | 0.60 | 0.24 | 0.24 | 0.54 | 0.59 | 0.63 | 0.80 | 0.18 | 0.51 | 0.60 | 0.66 | 0.45 | 0.79 | 0.22 | 0.27 | 0.37 |  |  |
|  |  |  | SD ^h^ | 0.08 | 0.25 | 0.04 | 0.35 | 0.32 | 0.41 | 0.14 | 0.14 | 0.31 | 0.35 | 0.33 | 0.34 | 0.17 | 0.27 | 0.17 | 0.32 |  |  |
| spinach ^b^ | Spinacia oleracea | 12 | AM ^g^ | 0.45 | 0.04 | 0.76 | 0.73 | 0.30 | 0.53 | 0.81 | 0.49 | 0.57 | 0.66 | 0.37 | 0.10 | 0.30 | 0.13 | 0.50 | 0.31 |  |  |
|  |  |  | SD ^h^ | 0.06 | 0.02 | 0.20 | 0.23 | 0.22 | 0.15 | 0.01 | 0.25 | 0.23 | 0.30 | 0.01 | 0.02 | 0.10 | 0.07 | 0.16 | 0.08 |  |  |
| coriander ^b^ | Coriandrum sativum | 43 | AM ^g^ | 0.16 | 0.03 | 0.71 | 0.32 | 0.39 | 0.57 | 0.53 | 0.41 | 0.57 | 0.47 | 0.10 | 0.31 | 0.64 | 0.01 | 0.70 | 0.54 |  |  |
|  |  |  | SD ^h^ | 0.20 | 0.01 | 0.16 | 0.20 | 0.23 | 0.15 | 0.19 | 0.28 | 0.26 | 0.18 | 0.02 | 0.16 | 0.18 | 0.01 | 0.26 | 0.30 |  |  |
| cauliflower ^d^ | Brassica oleracea B. | 33 | AM ^g^ | 0.46 | 0.05 | 0.71 | 0.19 | 0.36 | 0.53 | 0.70 | 0.28 | 0.17 | 0.59 | 0.42 | 0.44 | 0.60 | 0.50 | 0.46 | 0.47 |  |  |
|  |  |  | SD ^h^ | 0.12 | 0.02 | 0.17 | 0.23 | 0.32 | 0.16 | 0.17 | 0.12 | 0.13 | 0.19 | 0.26 | 0.21 | 0.17 | 0.21 | 0.28 | 0.28 |  |  |
| pea ^e^ | Pisum sativum | 31 | AM ^g^ | 0.58 | 0.04 | 0.19 | 0.35 | 0.16 | 0.57 | 0.52 | 0.22 | 0.39 | 0.38 | 0.46 | 0.37 | 0.52 | 0.38 | 0.33 | 0.37 |  |  |
|  |  |  | SD ^h^ | 0.19 | 0.03 | 0.09 | 0.27 | 0.08 | 0.19 | 0.17 | 0.23 | 0.22 | 0.21 | 0.26 | 0.35 | 0.28 | 0.26 | 0.17 | 0.23 |  |  |
| lettuce ^b^ | Lactuca sativa | 13 | AM ^g^ |  |  |  | 0.0013 |  |  |  |  |  |  |  |  | 0.0009 |  |  |  |  | [14] |
|  |  |  | SD ^h^ |  |  |  | 0.0008 |  |  |  |  |  |  |  |  | 0.0005 |  |  |  |  |  |
| squash ^e^ | Cucurbita pepo | 15 | AM ^g^ |  |  |  | 0.0182 |  |  |  |  |  |  |  |  | 0.0015 |  |  |  |  |  |
|  |  |  | SD ^h^ |  |  |  | 0.0260 |  |  |  |  |  |  |  |  | 0.0018 |  |  |  |  |  |
| potato w. peel ^f^ | Solanum tuberosum | 15 | AM ^g^ |  |  |  | 0.0349 |  |  |  |  |  |  |  |  | 0.0032 |  | 0.0037 |  |  |  |
|  |  |  | SD ^h^ |  |  |  | 0.0513 |  |  |  |  |  |  |  |  | 0.0023 |  | 0.0023 |  |  |  |
| carrot w. peel ^c^ | Daucus carota | 14 | AM ^g^ |  |  |  | 0.0108 |  |  |  |  |  |  |  |  | 0.0027 |  | 0.0042 |  |  |  |
|  |  |  | SD ^h^ |  |  |  | 0.0073 |  |  |  |  |  |  |  |  | 0.0019 |  | 0.0027 |  |  |  |

^a^ bulb vegetable.

^b^ leaf vegetable.

^c^ root vegetable.

^d^ flower vegetable.

^e^ fruit vegetable.

^f^ stem vegetable.

^g^ arithmetic mean.

^h^ standard deviation.

^i^ coefficient of variance.

.

**S8 Table. Rotated factor pattern for PAHs in bulbs (n=9).**

| PAHs | PC1 (62.70%) | PC2 (24.25%) |
| --- | --- | --- |
| Nap | 0.26 | 0.88 |
| Acy | -0.15 | 0.81 |
| Ace | 0.90 | 0.03 |
| Flu | 0.81 | 0.53 |
| Phe | 0.88 | 0.42 |
| Ant | 0.98 | 0.14 |
| Flt | 0.93 | -0.02 |
| Pyr | 0.93 | -0.14 |

Extraction Method: Principle Component Analysis.

Rotation Method: Varimax with Kaiser Normalization.

**S9 Table.** **Pearson correlation coefficients between the concentrations of PAHs in bulbs and those in soil.**

|  | **Nap** | **Acy** | **Ace** | **Flu** | **Phe** | **Ant** | **Flt** | **Pyr** | **∑PAH** |
| --- | --- | --- | --- | --- | --- | --- | --- | --- | --- |
| Nap | 0.49  (0.41) |  |  |  |  |  |  |  |  |
| Acy |  | 0.50  (0.40) |  |  |  |  |  |  |  |
| Ace |  |  | -0.05  (0.94) |  |  |  |  |  |  |
| Flu |  |  |  | 0.57  (0.32) |  |  |  |  |  |
| Phe |  |  |  |  | -0.20  (0.75) |  |  |  |  |
| Ant |  |  |  |  |  | 0.13  (0.83) |  |  |  |
| Flt |  |  |  |  |  |  | -0.90*  (0.04) |  |  |
| Pyr |  |  |  |  |  |  |  | -0.38  (0.53) |  |
| ∑PAH |  |  |  |  |  |  |  |  | -1.00  (0.08) |

**S10 Table. Concentrations of PAHs in baked bulbs (n=9).**

| **PAHs** | **Nap** | **Acy** | **Ace** | **Flu** | **Phe** | **Ant** | **Flt** | **Pyr** | **BaA** | **Chr** | **BbF** | **BkF** | **BaP** | **DahA** | **IP** | **BghiP** | **ΣPAHs** |
| --- | --- | --- | --- | --- | --- | --- | --- | --- | --- | --- | --- | --- | --- | --- | --- | --- | --- |
| AM ^a^ (μg kg^-1^, fw) | 275.33 | 2.58 | 2.61 | 44.93 | 134.28 | 3.90 | 16.38 | 12.63 | 1.40 | 5.30 | - | - | - | - | - | - | 488.51 |
| SD ^b^ | 117.27 | 2.37 | 0.75 | 14.81 | 41.17 | / | 5.92 | 3.14 | / | / | - | - | - | - | - | - | 172.42 |
| Median (μg kg^-1^, fw) | 255.00 | 1.70 | 2.90 | 47.40 | 145.00 | / | 17.50 | 11.60 | / | / | - | - | - | - | - | - | 507.80 |
| Max (μg kg^-1^, fw) | 473.00 | 8.20 | 3.80 | 69.70 | 192.00 | / | 24.50 | 18.10 | / | / | - | - | - | - | - | - | 743.00 |
| Min (μg kg^-1^, fw) | 166.00 | 1.20 | 1.50 | 25.90 | 79.20 | / | 5.10 | 9.40 | / | / | - | - | - | - | - | - | 300.80 |
| CV ^c^ (%) | 42.59 | 92.08 | 28.88 | 32.97 | 30.66 | / | 36.12 | 24.83 | / | / | - | - | - | - | - | - | 35.29 |

^a^ arithmetic mean.

^b^ standard deviation.

^c^ coefficient of variance.

fw, fresh weight.

-, not detected in raw bulb sample.

/, Ant, BaA and Chr were detected in only one baked bulb sample, so SD, Median, Max, Min and CV were not calculated.

**S11 Table. Incremental lifetime cancer risk (ILCR) values of PAHs in lily bulb before and after baking.**

| **PAHs** | **raw bulb** | | | **baked bulb** | | |
| --- | --- | --- | --- | --- | --- | --- |
|  | **10%** | **50%** | **90%** | **10%** | **50%** | **90%** |
| Nap | 3.23E-07 | 6.94E-07 | 1.13E-06 | 1.33E-06 | 2.97E-06 | 4.93E-06 |
| Acy | 4.67E-09 | 1.01E-08 | 2.21E-08 | -4.80E-09 | 2.81E-08 | 6.36E-08 |
| Ace | 3.99E-09 | 1.52E-08 | 2.77E-08 | 1.66E-08 | 2.80E-08 | 4.22E-08 |
| Flu | 5.23E-08 | 1.31E-07 | 2.24E-07 | 2.70E-07 | 4.87E-07 | 7.51E-07 |
| Phe | 1.77E-07 | 4.52E-07 | 7.82E-07 | 8.49E-07 | 1.45E-06 | 2.19E-06 |
| Ant | 2.48E-08 | 9.29E-08 | 1.71E-07 | 3.35E-07 | 4.25E-07 | 5.38E-07 |
| Flt | 1.59E-08 | 1.04E-07 | 2.01E-07 | 9.20E-08 | 1.76E-07 | 2.79E-07 |
| Pyr | -1.20E-08 | 1.37E-07 | 2.98E-07 | 8.75E-08 | 1.36E-07 | 1.96E-07 |
| BaA | 1.40E-06 | 1.77E-06 | 2.24E-06 | 1.20E-06 | 1.53E-06 | 1.93E-06 |
| Chr | 2.26E-07 | 2.87E-07 | 3.63E-07 | 4.55E-07 | 5.78E-07 | 7.32E-07 |
| ILCR | 2.82E-06 | 3.71E-06 | 4.85E-06 | 5.55E-06 | 7.81E-06 | 1.07E-05 |

**S1 Fig. The total PAH concentrations in each raw bulb sample.** The red line represents the median value of the total PAH concentrations.

**S2 Fig. Composition of 16 PAHs in raw lily bulb samples.**





**S3 Fig. Comparison of detected PAHs in raw and baked bulbs.**





**S4 Fig.** **PAH composition profiles in PM_2.5_ and the corresponding gaseous phase from chunk coal combustion obtained by field investigation.**





**S5 Fig.** **PAH composition profiles in PM_10_ and the corresponding gaseous phase from chunk coal combustion obtained by field investigation.**





**S6 Fig. Diagnostic ratios of Flt/(Flt + Pyr) for raw bulbs, baked bulbs, and emissions from domestic coal combustion.** Red dots represent measured values by field investigation. Green solid lines represent the arithmetic means of each group. PP: Particulate phase; GP: Gaseous phase.

**Reference**

1. Cao YX, Lin CY, Zhang X, Liu XT, He MC, Ouyang W. Distribution, source, and ecological risks of polycyclic aromatic hydrocarbons in Lake Qinghai, China. Environ. Pollut. 2020; 266: 115401. [doi: 10.1016/j.envpol.2020.115401](https://doi.org/10.1016/j.envpol.2020.115401).
2. Zhu Y, Huang HJ, Zhang YH, Xiong GN, Zhang QH, Li YJ, et al. Evaluation of PAHs in edible parts of vegetables and their human health risks in Jinzhong City, Shanxi Province, China: A multimedia modeling approach. Sci. Total Environ. 2021; 773: 145076. [doi: 10.1016/j.scitotenv.2021.145076](https://doi.org/10.1016/j.scitotenv.2021.145076).
3. Paris A, Ledauphin J, Poinot P, Gaillard JL. Polycyclic aromatic hydrocarbons in fruits and vegetables: Origin, analysis, and occurrence. Environ. Pollut. 2018; 234: 96–106. [doi: 10.1016/j.envpol.2017.11.028](https://doi.org/10.1016/j.envpol.2017.11.028).
4. Jia JP, Bi CJ, Zhang JF, Chen ZL. Atmospheric deposition and vegetable uptake of polycyclic aromatic hydrocarbons (PAHs) based on experimental and computational simulations. Atmos. Environ. 2019; 204: 135–141. [doi: 10.1016/j.atmosenv.2019.02.030](https://doi.org/10.1016/j.atmosenv.2019.02.030).
5. Nisbet ICT, LaGoy PK. Toxic equivalency factors (TEFs) for polycyclic aromatic hydrocarbons (PAHs). Regul. Toxicol. Pharmacol. 1992; 16: 290–300. [doi: 10.1016/0273-2300(92)90009-X](https://doi.org/10.1016/0273-2300(92)90009-X).
6. Li RQ, Yu AF, Bai B, Xu R, Ding WJ. Potential health and ecological risks of accumulation of cadmium, lead and mercury in soil-edible lily systems. Food Sci. 2016; 37: 186–191. doi: 10.7506/spkx1002-6630-201605033.
7. Duan XL. Environmental Exposure Related Activity Patterns Survey of Chinese Population (Adults). Beijing: Environmental Science Press; 2013.
8. Tian K, Bao HY, Zhang XC, Shi TR, Liu XP, Wu FY. Residuals, bioaccessibility and health risk assessment of PAHs in winter wheat grains from areas influenced by coal combustion in China. Sci. Total Environ. 2018; 618: 777–784. [doi: 10.1016/j.scitotenv.2017.08.174](https://doi.org/10.1016/j.scitotenv.2017.08.174).
9. Wang BB, Gao F, Qin N, Duan XL, Li YJ, Cao SZ. A comprehensive analysis on source-distribution-bioaccumulation-exposure risk of metal(loid)s in various vegetables in peri-urban areas of Shenzhen, China. Environ. Pollut. 2022; 293: 118613. doi: 10.1016/j.envpol.2021.118613.
10. Soceanu A, Dobrinas S, Stanciu G, Popescu V. Polycyclic aromatic hydrocarbons in vegetables grown in urban and rural areas. Environ. Eng. Manag. J. 2014; 13: 2311–2315.
11. Martorell I, Perelló G, Martí-Cid R, Castell V, Llobet JM, Domingo JL. Polycyclic aromatic hydrocarbons (PAH) in foods and estimated PAH intake by the population of Catalonia, Spain: Temporal trend. Environ. Int. 2010; 36: 424–432. [doi: 10.1016/j.envint.2010.03.003](https://doi.org/10.1016/j.envint.2010.03.003).
12. Waqas M, Khan S, Chao C, Shamshad I, Qamar Z, Khan K. Quantification of PAHs and health risk via ingestion of vegetable in Khyber Pakhtunkhwa Province, Pakistan. Sci. Total Environ. 2014; 497–498: 448–458. [doi: 10.1016/j.scitotenv.2014.07.128](https://doi.org/10.1016/j.scitotenv.2014.07.128).
13. Chen YN, Zhang F, Zhang JQ, Zhou M, Li FX, Liu XP. Accumulation characteristics and potential risk of PAHs in vegetable system grow in home garden under straw burning condition in Jilin, Northeast China. Ecotoxicol. Environ. Saf. 2018; 162: 647–654. [doi: 10.1016/j.ecoenv.2018.06.082](https://doi.org/10.1016/j.ecoenv.2018.06.082).
14. Samsøe-Petersen L, Larsen EH, Larsen PB, Bruun P. Uptake of trace elements and PAHs by fruit and vegetables from contaminated soils. Environ. Sci. Technol. 2002; 36: 3057–3063. [doi: 10.1021/es015691t](https://doi.org/10.1021/es015691t).
